# Differences in Left Ventricular Remodeling Between Bicuspid Compared to Tricuspid Aortic Valve With Aortic Stenosis in a Chinese Population After Transcatheter Aortic Valve Replacement

**DOI:** 10.31083/RCM44165

**Published:** 2025-12-24

**Authors:** Chengwei Chi, Jiaqi Zhang, Weilong Zhao, Yuwei Wang, Yuxin Shao, Qingtao Meng, Shulong Zhang, Jiyi Liu, Simiao Tian, Jihong Liu

**Affiliations:** ^1^Department of Cardiology, Affiliated Zhongshan Hospital of Dalian University, 116001 Dalian, Liaoning, China; ^2^Cardiovascular Medical Department, The Fourth Affiliated Hospital of Harbin Medical University, 150001 Harbin, Heilongjiang, China

**Keywords:** aortic valve, transcatheter aortic valve replacement, left ventricular remodeling, echocardiography

## Abstract

**Background:**

The study aimed to compare the differences in reverse left ventricular (LV) remodeling following transcatheter aortic valve replacement (TAVR) between patients with the bicuspid aortic valve (BAV) and those with tricuspid aortic valve (TAV), both with aortic stenosis, in a Chinese population.

**Methods:**

A total of 137 patients were enrolled who were treated with a self-expandable Venus A valve at our center, who underwent TAVR from January 1, 2019, to June 30, 2022. We retrospectively included patients with BAV and TAV who underwent echocardiographic follow-ups at baseline and at least 6 months after the procedure.

**Results:**

Patients with a BAV were younger than those with a TAV. The BAV patients had a larger aortic root diameter (ARD), although the size of valve implantation was comparable between the two groups. Patients with a BAV might experience less reverse LV remodeling post-TAVR than patients with a TAV during the one-month follow-up (140.09 ± 36.94 g/m^2^ vs. 126.36 ± 26.96 g/m^2^; *p* = 0.044). There were no significant differences in the LV mass index (LVMi) between the two groups throughout the 24 hours or the six-month follow-up post-TAVR. Patients with a higher mean pressure gradient (MPG) (95% confidence interval (CI): 0.112–0.581; *p* = 0.004) and a larger ARD (95% CI: 0.519–5.573; *p* = 0.019) before TAVR had favorable mid-term LV reverse remodeling (ΔLVMi within 6 months) post-TAVR. Patients with much more severe aortic stenosis (AS) had favorable mid-term LV reverse remodeling post-TAVR.

**Conclusions:**

Patients with BAV might experience less reverse LV remodeling post-TAVR than patients with TAV during a short-term follow-up, but similar remodeling during mid-term follow-ups.

## 1. Introduction

As one of the most common congenital cardiac valve abnormalities, bicuspid aortic valve (BAV) disease has drawn our attention gradually. The prevalence of BAV is estimated to be 1%–2% of the general population [1]. The BAV is prone to progression through aortic valve stenosis (AVS) as well as aortic valve regurgitation due to calcific degeneration and accelerated sclerosis compared to the tricuspid aortic valve (TAV) [2]. Severe symptomatic BAV with aortic stenosis requiring intervention. Transcatheter aortic valve replacement (TAVR) has become a well-established therapy of choice for patients with severe AVS, who are at high risk for surgical aortic valve replacement [3]. However, the BAV was deemed to be a relative contraindication for TAVR because of anatomical differences rather than the TAVR. Even if experienced cardiac operators begin to decrease the risk of TAVR procedures, they still face this challenge [4]. Therefore, many patients with BAV were excluded from most randomized trials of TAVR procedures [5, 6]. An increasing number of centers around the world have started to perform TAVR on BAV patients [7, 8, 9, 10, 11, 12]. Given that China has a relatively high prevalence of BAV, investigating ventricular reverse remodeling and its prognostic implications after TAVR in this population is of particular importance.

The question of what outcome of TAVR in patients with BAV arises. Generally, BAV patients have more asymmetrical aortic valve calcification, larger annular dimensions, and more irregular and annular eccentricity, which may result in altered aortic geometry and blood flow [13, 14]. Geometric mismatching between a circular valve prosthesis and an asymmetric calcified ovoid annulus may lead to leaflet asymmetry or paravalvular leakage (PVL). These findings are related to the potential increased risk of aortic annular rupture and aortic dissection [13]. The use of the TAVR in treating BAV has been reported in several cases referring to decreased TAVR success rate, increased PVL, increased risk and rate of permanent pacemaker implantation, and worsened improvement in hemodynamics [15, 16, 17, 18, 19]. A higher incidence of major adverse cardiovascular events, especially excessive mortality, was reported in one case series [20]. Nevertheless, several large studies have concluded that the difference in outcomes after TAVR between BAV and TAV has no statistical significance nowadays [1, 7, 8, 21, 22, 23].

Left ventricular (LV) remodeling has been recognized as an important determinant of prognosis after TAVR. Favorable reverse remodeling is associated with symptomatic improvement, enhanced cardiac function, and better long-term outcomes, whereas inadequate remodeling may predict adverse events and reduced survival. Despite its clinical importance, data directly comparing post-TAVR ventricular remodeling between BAV and TAV patients remain scarce, particularly in the Chinese population. Several studies have shown differences in the use of the TAVR between the BAV and TAV groups in Western populations [16, 24]. However, limited data describing the outcomes of TAVR in Chinese patients with bicuspid aortic stenosis versus tricuspid aortic stenosis exist.

Therefore, the present study aimed to comprehensively evaluate the efficacy and outcomes of TAVR with a self-expanding valve in patients with BAV compared with those with TAV, both with aortic stenosis. In particular, we sought to investigate the temporal differences in LV reverse remodeling between BAV and TAV patients during short-term and mid-term follow-up. Furthermore, we aimed to explore the potential prognostic implications of these remodeling patterns, with the goal of providing novel insights for optimizing therapeutic strategies, refining patient selection, and improving risk stratification in this heterogeneous patient population.

## 2. Materials and Methods

### 2.1 Study Population

#### 2.1.1 Patients Selection

137 patients with severe symptomatic AVS who received TAVR via the self-expandable valve at our center from January 1, 2019 to June 30, 2022 were enrolled in this study. We accessed data for research purposes in January 1, 2024. The indications for selected patients for TAVR were thoroughly discussed by our multidisciplinary heart team, which included cardiologists, cardiac surgeons, echocardiologists, cardiac radiologists, cardiac interventional physicians, anesthetists, and doctors involved in cardiopulmonary bypass. Every echocardiographic examination was performed by one experienced echocardiologist.

#### 2.1.2 Inclusion Criteria

The key inclusion criteria were as follows: (1) severe AS patients diagnosed on the recommendation of the European Society of Cardiology/European Association for Cardio-Thoracic Surgery Guidelines (aortic valve area [AVA] ≤1.0 cm^2^/aortic valve index ≤0.6 cm^2^/m^2^/peak aortic velocity (Vmax) ≥4.0 m/s); (2) patients at intermediate- to high-risk surgical risk or a Society of Thoracic Surgeons risk (STS) score >4; and (3) patients with severe AS with typical symptoms.

#### 2.1.3 Exclusion Criteria

**Fig. 1.**
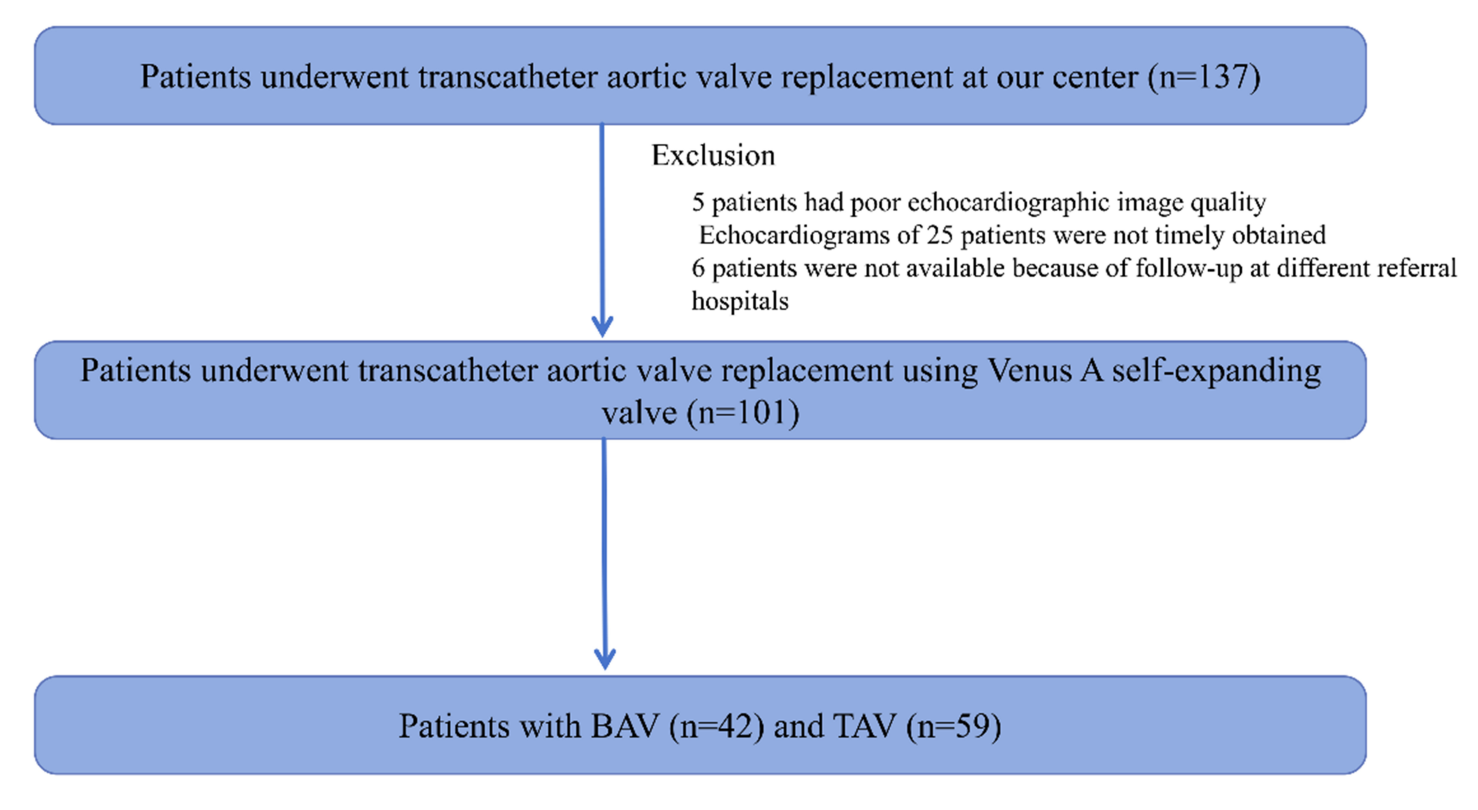
**Study flow chart**. BAV, bicuspid aortic valve; TAV, tricuspid aortic valve.

The main exclusion criteria were as follows: (1) patients with active endocarditis, acute aortic dissection, or acute myocardial infarction; (2) patients with expectations of life <1 year. Patients who had poor echocardiographic image quality (n = 5) during follow-up and who underwent follow-up at another referral hospital (n = 6) were excluded. Echocardiograms of 25 patients were not obtained in a timely manner after TAVR during the six-month follow-up, as shown in Fig. 1. Considering this, 101 patients fulfilled the criteria of this study and were divided into two groups (BAV: 42; TAV: 59). The type of aortic valve was defined on the basis of the number of cusps, the number of raphes and the spatial orientation of the raphe [25]. The morphology and annulus dimensions of the aortic valve were confirmed through echocardiography and multidetector computed tomography (MDCT).

### 2.2 Echocardiography

All patients in the two groups underwent echocardiography at baseline, before TAVR, 24 hours after TAVR, at one month after TAVR and at the six-month follow-up. All echocardiograms were performed by the same echocardiologist on a Phillips EPIQ 7 system equipped with a 2.5-MHz transducer. All the data, which were stored digitally, were analyzed by the same echocardiologist. The operator was completely blinded to all patients. Two-dimensional echocardiograms were used to measure the left ventricular end diastole diameter (LVEDD), interventricular septum in diastole (IVSD), and left ventricular posterior wall thickness (LVPWT). Other echocardiographic data including the mean pressure gradient (MPG) and AVA, were also collected. The Devereux formula highly recommended by the American Society of Echocardiography was used to calculate the left ventricular mass (LVM). Body surface area (BSA) and LVM were used to calculate the left ventricular mass index (LVMi). BSA indexing is weight sensitive and height sensitive, especially for LVM. The relative wall thickness (RWT) was calculated as (LVPWT × 2/LVEDD. The formula used was LVM (g) = 0.8 × 1.04 × [(LVEDD + IVSD + LVPWT)^3^ − LVEDD^3^] + 0.6. LVMi was calculated with the formula LVM/BSA. The LV ejection fraction (LVEF) was calculated based on Simpson’s method (apical 2,4-chamber views). The definition of LV hypertrophy (LVH) was LVMi exceeding 115 g/m^2^ in men and 95 g/m^2^ in women.

### 2.3 TAVR Procedure

The transfemoral TAVR was the treatment of choice. However, an alternate appropriate vascular access was used when the transfemoral approach (TF) was infeasible because of the small diameter, high tortuosity, and severe calcification. In accordance with the anatomy of the femoral vessels and iliac region or history of peripheral artery disease (PAD), an appropriate approach involving the use of the TAVR was employed, including the TF, transapical approach (TA), or transaxillary approach (TAX). A Venus A valve (Venus Med Tech, Inc., Hangzhou, China) was used in this study. All patients underwent the TAVR procedure under general anesthesia. The type of prosthesis was selected based on its usability at the time of the TAVR procedure. The size of the valve was determined in accordance with the comprehensive analysis of landing zone calcification as well as annular dimensions. For balloon pre-dilation or post-dilation, devices were used on basis of operator discretion. For the most part, a root angiogram was performed prior to balloon deployment. Briefly, pre-dilation was routinely conducted with the exception of calcification. An additional valve was implanted when moderate or severe aortic regurgitation occurred after balloon deployment. The PVL after TAVR was assessed via echocardiography.

### 2.4 Statistical Analysis

All continuous data are expressed as the means ± standard deviations (SD) or medians ± range, as appropriate, whereas categorical variables are expressed as numbers and percentages. Categorical variables were compared using Chi-squared or Fisher’s exact tests as appropriate. Differences between means were evaluated using paired (for before and after comparisons) and unpaired (for independent group comparisons) Student’s *t*-test for normally distributed data and Mann-Whitney or Wilcoxon signed-rank tests for nonparametric data. “Mid-term” was defined as referring to the echocardiographic assessment of LV structure and function at 6 months after TAVR. To identify factors associated with mid-term regression of the LVMi at 6-month follow-up, multivariable linear regression, with Firth’s correction due to the small sample size, was performed. This statistical method allowed us to identify independent predictors of LVMi changes after TAVR while adjusting for potential confounding factors. Graph generations were performed using GraphPad Prism version 8.0 (GraphPad Software, San Diego, CA, USA). SPSS Version 25.0 (Armonk, NY, USA: IBM Corp.) was used for data analysis, and a *p*-value < 0.05 was considered to indicate statistical significance.

## 3. Results

### 3.1 Patient Baseline Characteristics

In total, 101 patients who had detailed baseline characteristics (mean age 74.50 ± 8.32 years, 41.58% BAV) were enrolled in our analysis, as shown in Table 1. All the patients underwent a TAVR procedure; 42 patients had BAV, and 59 patients had TAV. At baseline, BAV patients who underwent TAVR were significantly younger than TAV patients (0.71 ± 7.60 years vs. 77.20 ± 7.78 years; *p* < 0.001). Patients with TAV exhibited a higher prevalence of CAD, which reached statistical significance (*p* < 0.05). The EuroScore II score, the mean STS mortality score and the STS Morbidity-Mortality score were significantly different at baseline. The three types of scores above were much lower for the BAV patients than for the TAV patients (*p* < 0.05).

**Table 1.** **Baseline characteristics**.

		All (n = 101)	BAV (n = 42)	TAV (n = 59)	*p*-value
Age (years, $\bar{x}$ ± *s*)		74.50 ± 8.32	70.71 ± 7.60	77.20 ± 7.78	<0.001
BSA (m^2^, $\bar{x}$ ± *s*)		1.78 ± 0.18	1.78 ± 0.18	1.78 ± 0.18	0.923
Male		48 (47.52)	22 (52.38)	26 (44.07)	0.427
NYHA class III/IV		81 (80.20)	33 (78.57)	48 (81.36)	0.802
CrCl (mL/minute, $\bar{x}$ ± *s*)		79.55 ± 35.42	84.86 ± 32.52	75.77 ± 37.15	0.205
Comorbidities					
	Hypertension	63 (62.38)	22 (52.38)	41 (69.49)	0.097
	Dyslipidemia	60 (59.41)	26 (61.90)	34 (57.63)	0.687
	Diabetes	37 (36.63)	15 (35.71)	22 (37.29)	1.000
	COPD	5 (4.95)	1 (2.38)	4 (6.78)	0.590
	Stroke	43 (42.57)	18 (42.86)	25 (42.37)	1.000
	PVD	30 (29.70)	9 (21.43)	21 (35.59)	0.185
	CAD	58 (57.43)	19 (45.24)	39 (66.10)	0.043
	Atrial fibrillation	31 (30.69)	10 (23.81)	21 (35.59)	0.274
	Previous valvular replacement surgery	3 (2.970)	2 (4.76)	1 (1.69)	0.764
	prior CABG	2 (1.980)	0	2 (3.39)	0.509
	Need for urgent aortic valvular intervention	2 (1.980)	1 (2.38)	1 (1.69)	1.000
	EuroScore II ( $\bar{x}$ ± *s*)	6.83 ± 6.95	4.39 ± 3.67	8.57 ± 8.15	0.001
	STS Mortality ($\bar{x}$ ± *s*)	3.97 ± 3.37	2.60 ± 2.04	4.94 ± 3.78	<0.001
	STS Morbimortality ($\bar{x}$ ± *s*)	14.54 ± 7.57	11.67 ± 6.21	16.59 ± 7.84	0.001
Electrocardiogram					
	Sinus	79 (78.22)	36 (85.71)	43 (72.88)	0.148
	Atrial fibrillation	18 (17.82)	5 (11.90)	13 (22.03)	0.291
	Other atrial rhythm	4 (3.96)	2 (4.76)	2 (3.38)	1.000
	Abnormal cardiac electric axis	56 (55.45)	23 (54.76)	33 (55.93)	1.000
	1° AVB	16 (15.84)	9 (21.43)	7 (11.86)	0.269
	LBBB	4 (3.96)	2 (4.76)	2 (3.39)	1.000
	RBBB	12 (11.88)	6 (14.29)	6 (10.16)	0.750
	LAFB	5 (4.95)	3 (7.14)	2 (3.39)	0.695
Echocardiogram					
	MPG (mmHg, $\bar{x}$ ± *s*)	50.19 ± 24.21	60.10 ± 29.34	43.14 ± 16.75	0.001
	AVA (cm^2^, $\bar{x}$ ± *s*)	0.70 ± 0.22	0.67 ± 0.18	0.72 ± 0.24	0.196
	AVA/BSA (cm^2^/m^2^, $\bar{x}$ ± *s*)	0.39 ± 0.12	0.38 ± 0.10	0.41 ± 0.14	0.212
	LVEF (%, $\bar{x}$ ± *s*)	52.23 ± 12.42	52.07 ± 12.63	52.34 ± 12.38	0.916
	LVEDD (mm, $\bar{x}$ ± *s*)	51.44 ± 8.02	50.55 ± 8.41	52.07 ± 7.74	0.351
	ARD (mm, $\bar{x}$ ± *s*)	20.44 ± 2.55	20.79 ± 2.48	20.19 ± 2.58	0.246
	LVPWT (mm, $\bar{x}$ ± *s*)	12.12 ± 2.13	12.76 ± 2.20	11.66 ± 1.97	0.010
	IVSD (mm, $\bar{x}$ ± *s*)	13.34 ± 2.44	13.95 ± 2.78	12.90 ± 2.08	0.041
	LVMi (g/m^2^, $\bar{x}$ ± *s*)	151.17 ± 41.37	158.36 ± 46.54	146.05 ± 36.82	0.141
	RWT ($\bar{x}$ ± *s*)	0.51 ± 0.13	0.55 ± 0.14	0.49 ± 0.12	0.022

BSA, body surface area; NYHA, New York Heart Association; CrCl, creatinine clearance; COPD, chronic obstructive pulmonary disease; PVD, peripheral vascular disease; CAD, coronary artery disease; CABG, coronary artery bypass graft; STS, Society of Thoracic Surgeons; 1°AVB, first-degree atrioventricular block; LBBB, left bundle branch block; RBBB, right bundle branch block; LAFB, left anterior fascicular block; MPG, mean aortic pressure gradient; AVA, aortic valvular area; LVEF, left ventricular ejection fraction; LVEDD, left ventricular end-diastolic dimension; ARD, aortic root diameter; LVPWT, left ventricular posterior wall thickness; IVSD, interventricular septum in diastole; LVMi, left ventricular mass index; RWT, relative wall thickness; *p*-values in bold are statistically significant.

### 3.2 Pre-TAVR Echocardiographic Characteristics

The baseline echocardiographic data showed no differences except for the MPG, LVPWT, IVSD and RWT. The MPG, LVPWT, IVSD and RWT were observed to be much higher in the BAV than in the TAV, as shown in Table 1 (*p* < 0.05).

### 3.3 Procedural Characteristics and Outcomes

As shown in Table 2, compared with those of TAV patients, the size of the valve implanted in BAV patients was comparable in Table 2. The size of aortic annulus diameter (AOD) in BAV group was much bigger than that in TAV group (23.92 ± 3.14 mm vs. 24.68 ± 3.98 mm; *p* = 0.038). Moderate or severe PVL was observed in 4 patients (9.52%) in the BAV group and in 3 patients (5.08%) in the TAV group, with no statistically significant difference between the two groups (*p* = 0.640).

**Table 2.** **Procedural data**.

		All (n = 101)	BAV (n = 42)	TAV (n = 59)	*p*-value
Venus-A valve size					0.312
	23 mm	24 (23.76)	13 (30.95)	11 (18.64)	
	26 mm	53 (52.47)	19 (45.24)	34 (57.63)	
	29 mm	22 (21.78)	10 (23.81)	12 (20.34)	
	32 mm	2 (1.98)	0	2 (3.39)	
AOD (mm, $\bar{x}$ ± *s*)		23.92 ± 3.14	24.68 ± 3.98	23.37 ± 2.26	0.038
Mild paravalvular leak		7 (6.93)	2 (4.76)	5 (8.47)	0.744
Moderate/severe paravalvular leak		7 (6.93)	4 (9.52)	3 (5.08)	0.640

AOD, aortic annulus diameter *p*-values in bold are statistically significant.

### 3.4 Post-TAVR Echocardiographic Characteristics

As shown in Table 3, the improvements in the MPG, AVA, valvular aortic area indexed to the body surface area (AVA/BSA), left ventricular ejection fraction (LVEF), LVPWT, IVSD and LVMi in the BAV were statistically significant compared to the preprocedural values (*p* < 0.05). Additionally, significant decreases in the LVEDD were found in the BAV during the six-month follow-up (*p* < 0.05).

**Table 3.** **Echocardiographic characteristics: before the procedure, at discharge, at one-month follow-up and at six-month follow-up**.

		BAV (n = 42)	TAV (n = 59)	*p*-value
MPG (mmHg, $\bar{x}$ ± *s*)				
	Baseline	60.10 ± 29.34	43.14 ± 16.75	0.001
	24 hours after TAVR	17.98 ± 8.39^a^	10.71 ± 4.74^a^	<0.001
	1 M follow-up	16.14 ± 7.02^a^	10.83 ± 4.55^a^	<0.001
	6 M follow-up	15.38 ± 5.92^a^	11.08 ± 4.49^a^	<0.001
AVA (cm^2^, $\bar{x}$ ± *s*)				
	Baseline	0.67 ± 0.18	0.72 ± 0.24	0.196
	24 hours after TAVR	1.57 ± 0.44^a^	1.56 ± 0.37^a^	0.919
	1 M follow-up	1.59 ± 0.36^a^	1.64 ± 0.36^a^	0.496
	6 M follow-up	1.62 ± 0.31^a^	1.67±0.37^a^	0.403
AVA/BSA (cm^2^/m^2^, $\bar{x}$ ± *s*)				
	Baseline	0.38 ± 0.10	0.41 ± 0.14	0.212
	24 hours after TAVR	0.88 ± 0.20^a^	0.88 ± 0.18^a^	0.943
	1 M follow-up	0.91 ± 0.20^a^	0.92 ± 0.18^a^	0.716
	6 M follow-up	0.92 ± 0.16^a^	0.94 ± 0.18^a^	0.721
LVEF (%, $\bar{x}$ ± *s*)				
	Baseline	52.07 ± 12.63	52.34 ± 12.38	0.916
	24 hours after TAVR	55.29 ± 9.14^a^	52.75 ± 9.71^a^	0.537
	1 M follow-up	54.96 ± 8.73^a^	54.10 ± 9.69^a^	0.625
	6 M follow-up	58.69 ± 6.33^a^	57.20 ± 7.75^a^	0.308
LVEDD (mm, $\bar{x}$ ± *s*)				
	Baseline	50.55 ± 8.41	52.07 ± 7.74	0.351
	24 hours after TAVR	50.38 ± 8.21	50.61 ± 6.83^a^	0.879
	1 M follow-up	49.38 ± 7.51	49.59 ± 6.32^a^	0.878
	6 M follow-up	48.02 ± 7.20^a^	48.98 ± 6.56^a^	0.489
ARD (mm, $\bar{x}$ ± *s*)				
	Baseline	20.79 ± 2.48	20.19 ± 2.58	0.246
	24 hours after TAVR	20.52 ± 3.02	19.71 ± 2.67	0.157
	1 M follow-up	20.64 ± 3.19	19.56 ± 2.10	0.042
	6 M follow-up	20.14 ± 2.98	19.29 ± 1.99^a^	0.087
LVPWT (mm, $\bar{x}$ ± *s*)				
	Baseline	12.76 ± 2.20	11.66 ± 1.97	0.010
	24 hours after TAVR	12.33 ± 1.78^a^	11.49 ± 1.74	0.019
	1 M follow-up	12.14 ± 1.75^a^	11.25 ± 1.42^a^	0.006
	6 M follow-up	11.74 ± 1.52^a^	10.97 ± 1.23^a^	0.006
IVSD (mm, $\bar{x}$ ± *s*)				
	Baseline	13.95 ± 2.78	12.90 ± 2.08	0.041
	24 hours after TAVR	13.48 ± 2.23^a^	12.75 ± 2.02	0.090
	1 M follow-up	13.05 ± 2.04^a^	12.17 ± 1.69^a^	0.020
	6 M follow-up	12.57 ± 2.04^a^	11.92 ± 1.47^a^	0.079
LVMi (g/m^2^, $\bar{x}$ ± *s*)				
	Baseline	158.36 ± 46.54	146.05 ± 36.82	0.141
	24 hours after TAVR	149.14 ± 38.10^a^	136.94 ± 31.82^a^	0.083
	1 M follow-up	140.09 ± 36.94^a^	126.36 ± 26.96^a^	0.044
	6 M follow-up	127.39 ± 33.29^a^	119.91 ± 25.65^a^	0.205
RWT ($\bar{x}$ ± *s*)				
	Baseline	0.55 ± 0.14	0.49 ± 0.12	0.022
	24 hours after TAVR	0.53 ± 0.13	0.49 ± 0.11	0.104
	1 M follow-up	0.52 ± 0.11	0.48 ± 0.10	0.055
	6 M follow-up	0.52 ± 0.10	0.48 ± 0.09	0.043

TAVR, transcatheter aortic valve replacement; AVA, aortic valvular area; ^a^*p* < 0.05, *p*-values in bold are statistically significant.

Furthermore, the improvements in the MPG, AVA, AVA/BSA, LVEF, LVEDD and LVMi in patients with TAV were statistically significant during follow-up than before the procedure (*p* < 0.05). The improvements in the AOD in the TAV during the six-month follow-up were statistically significant compared to the preprocedural values (*p* < 0.05). Significant decreases of the TAV were found in LVPWT and IVSD during the first month and six-month follow-up (*p* < 0.05).

**Fig. 2.**
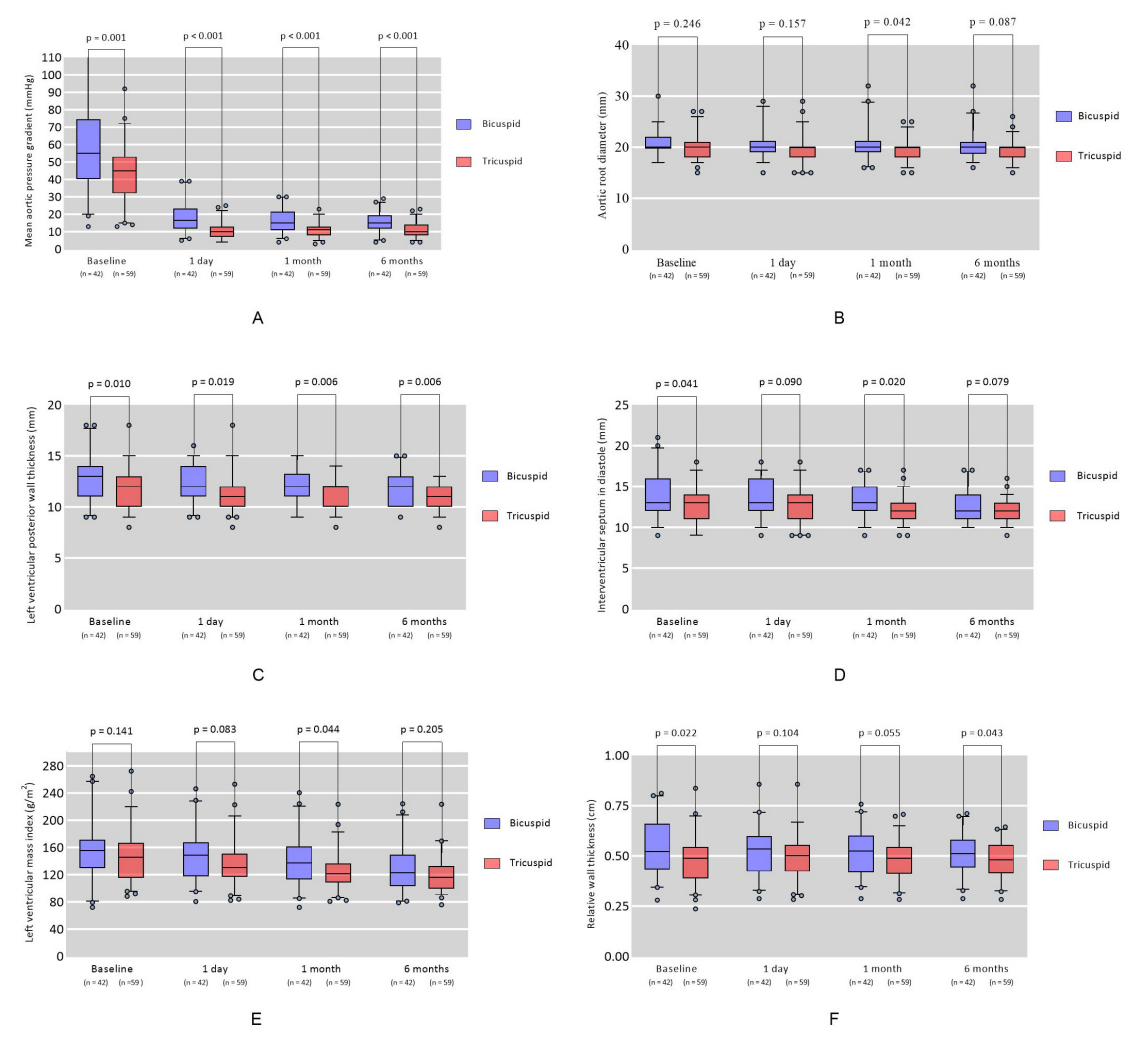
**Changes in mean aortic gradient (A), ARD (B), LVPWT (C), IVSD (D), LVMi (E), and relative wall thickness (RWT) (F), at six-month post-TAVR from baseline value in patients with bicuspid compared with tricuspid morphology**.

As shown in Table 3, the MPG in patients with BAV was 60.10 ± 29.34 mmHg, 17.98 ± 8.39 mmHg, 16.14 ± 7.02 mmHg, and 15.38 ± 5.92 mmHg pre-TAVR, at 24 hours, at first- and 6-month follow-up. The MPG and LVPWT were higher in BAV patients than in TAV patients during each follow-up period (*p* < 0.05). Compared to that in patients with TAV, the AOD in patients with BAV during the first-month follow-up was larger (20.64 ± 3.19 mm vs. 19.56 ± 2.10 mm; *p* = 0.042). Larger LVMi in the BAV than in the TAV was found at the first month in Table 3 (140.09 ± 36.94 g/m^2^ vs. 126.36 ± 26.96 g/m^2^; *p* = 0.044). Compared to that in patients with TAV, the IVSD in patients with BAV during the first-month follow-up was larger (13.05 ± 2.04 mm vs. 12.17 ± 1.69 mm; *p* = 0.020). Significant improvements in the RWT during the baseline and six-month follow-up were found in patients with BAV than in those with TAV (at six-month follow-up: 0.52 ± 0.10 cm vs. 0.48 ± 0.09 cm; *p* = 0.043). A decrease in the MPG and LVPWT was found to be statistically significant during the postoperative follow-up, as shown in Fig. 2 (*p* < 0.05).

### 3.5 LV Reverse Remodeling at 6-Month Follow-Up

**Fig. 3.**
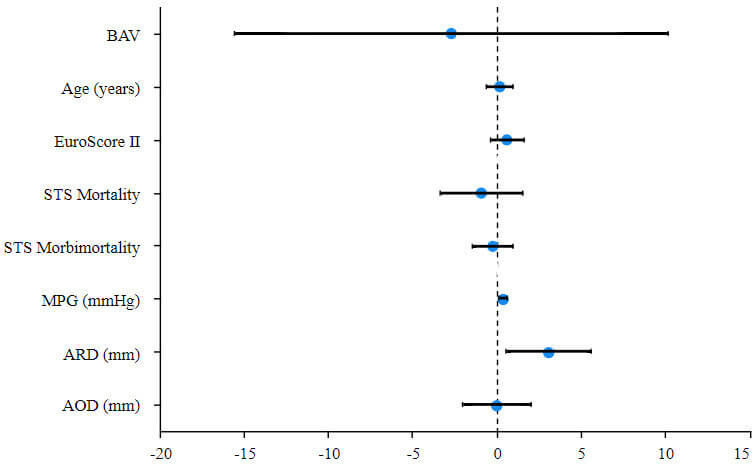
**Factors associated with mid-term regression of LVMi (ΔLVMi within 6 months) after TAVR**.

As shown in Table 4, the results of the multivariable linear regression were analyzed to assess the independent predictors of greater mid-term LV mass regression after TAVR. After adjusting for BAV, EuroScore Ⅱ, STS Mortality, MPG, aortic root diameter (ARD), AOD, aortic annulus diameter, multivariable linear-regression analysis demonstrated only the higher MPG (95% confidence interval [CI]: 0.112 to 0.581, *p* = 0.004) and bigger ARD (95% CI: 0.519 to 5.573, *p* = 0.019) prior to TAVR were independently associated with greater mid-term LVMi regression post-TAVR, as shown in Fig. 3.

**Table 4.** **Factors associated with mid-term regression of the LVMi at 6-month follow-up**.

	Model 1			Model 2		
	Beta	95% CI	*p*-value	Beta	95% CI	*p*-value
BAV	–4.826	–15.896 to 6.244	0.389	–3.432	–16.034 to 9.170	0.590
Age (years)	–0.183	–0.844 to 0.477	0.583	0.105	–0.687 to 0.897	0.793
EuroScore II	0.042	–0.749 to 0.834	0.916	0.556	–0.453 to 1.564	0.277
STS Mortality	–0.385	–2.018 to 1.248	0.641	–0.967	–3.435 to 1.500	0.438
STS Morbimortality	–0.256	–0.981 to 0.469	0.485	–0.272	–1.445 to 0.901	0.646
MPG (mmHg)	0.310	0.091 to 0.529	0.006	0.347	0.112 to 0.581	0.004
ARD (mm)	2.431	0.325 to 4.537	0.024	3.046	0.519 to 5.573	0.019
AOD (mm)	1.082	–0.655 to 2.819	0.219	–0.037	–2.072 to 1.997	0.971

Model 1. Crude analysis; Model 2. Adjusted for BAV, Age, EuroScore Ⅱ, STS Mortality, STS Morbimortality. *p*-values in bold are statistically significant; CI, confidence interval.

## 4. Discussion

The present study mainly characterized the further detailed outcomes of patients who underwent TAVR for BAV versus TAV for up to one year. Our observational study demonstrated that (1) the mean age of BAV patients who underwent TAVR were significantly younger compared to TAV patients; (2) the three types of risk scores and the prevalence of CAD were much lower for the BAV patients than for the TAV patients; (3) MPG, LVPWT, IVSD and RWT at baseline were observed much higher in BAV than TAV; (4) BAV patients were associated with bigger size of aortic annulus diameter, although the size of valve implantation was not significantly different between the two groups; (5) patients with BAV might experience less reverse left ventricle remodeling post-TAVR than patients with TAV during one-month follow-up. Moreover, there were no significant differences in the LVMi between the TAV and BAV throughout the 24-hour or six-month follow-up; (6) Patients with higher MPG and bigger AOD prior to TAVR had favorable mid-term LV reverse remodeling (ΔLVMi within six months) post-TAVR. Patients with much more severe AS had favorable mid-term LV reverse remodeling post-TAVR.

### 4.1 Clinical and Epidemiological Characteristics of BAV Patients Undergoing TAVR

In our study, the mean age of the BAV patients who underwent TAVR was 7 years younger than that of the TAV patients. Our findings demonstrated that patients with BAV had a lower prevalence of CAD and lower calculated risk scores compared with those with TAV. This is expected because symptomatic severe aortic valve regurgitation is more likely to rapidly develop at a younger age in patients with bicuspid morphology [1]. We have reason to assume that these differences are mainly age related, since patients in the BAV group developed aortic stenosis at a significantly younger age compared with those in the TAV group. Considering that age is a major determinant of both coronary atherosclerosis and surgical risk estimation, this age difference may explain the observed variation between the two groups. In our cohort, patients with BAV who underwent TAVR were on average 7 years younger than those with TAV. This significant age difference should be taken into account when interpreting the differences in left ventricular remodeling between the two groups. Age is a well-recognized determinant of LV compliance, with younger patients generally exhibiting more favorable myocardial elasticity and remodeling capacity. Therefore, the observed differences in reverse remodeling after TAVR may, at least in part, be explained by the age-related variations in LV compliance rather than by valve morphology alone. As one of the most common congenital cardiac valve abnormalities, BAV can be found in approximately 50% to 75% of patients with congenital aorta coarctation abnormalities [26, 27, 28]. Tzemos *et al*. [21] first demonstrated that almost half of the adult population with BAV had been diagnosed with moderate or severe aortic stenosis or regurgitation. Detaint *et al*. [1] reported that aortic valve surgery or any other cardiovascular surgery was required for BAV at a younger age than for TAV in the adult population.

### 4.2 Anatomical and Morphological Considerations in BAV

The size of AOD in the BAV cohort that we chose to use was larger than that in the TAV cohort during the procedure, although the size of valve implantation was not significantly different between the two groups. BAV patients who undergo TAVR tend to be younger and have larger size of aortic annulus areas and aorta diameters, as well as visible eccentric annular calcification [29]. Furthermore, BAV patients present unique challenges, as their more elliptical and asymmetric annulus can hinder optimal valve positioning, anchoring, and expansion [29]. Not infrequently in BAV, less fibrin content in the aortic wall leads to elastic fragmentation, increased collagen stiffness as well as altered expression of matrix metalloproteinases [30].

Sievers *et al*. [25] proposed a classification system based on the number of raphes and cusps coupled with their orientation about BAV morphology. Transthoracic echocardiography has a lower sensitivity than other methods and might miss some elderly patients with BAV referred for TAVR. Thus, a novel classification system that is TAVR-directed as well as simplified nonnumerical on the basis of leaflet orientation and heterogeneous leaflet morphologies exists [31]. MDCT assessment is mandatory when the TAVR procedure is planned. MDCT assessment is considered routine for detecting aortic annular sizing and is strongly correlated with intraoperative sizing during TAVR.

### 4.3 Coexistence of Aortic Stenosis and Transthyretin Amyloid Cardiomyopathy

AS and transthyretin-related amyloid cardiomyopathy (ATTR-CM) frequently coexist in elderly patients, especially men, with ATTR-CM reported in 4%–16% of AS cases [32]. Both conditions share risk factors such as advanced age and male sex, and often present with overlapping features, including HFpEF, increased LV wall thickness, and low-flow, low-gradient AS [33]. ATTR-CM, caused by deposition of misfolded transthyretin fibrils, exists as wild-type (ATTRwt) or hereditary (ATTRv) forms [32, 33, 34]. While ATTR-CM does not directly induce AS, amyloid deposition in valvular tissue may contribute to fibrosis and calcification, and AS-related pressure overload may further accelerate myocardial amyloid deposition, creating a vicious cycle [34]. Clinically, patients with concurrent AS and ATTR-CM, particularly males, experience poorer outcomes after valve replacement, including persistent heart failure, limited reverse remodeling, and increased mortality. Although data are limited in the Chinese population, aging demographics suggest ATTR-CM may be underdiagnosed, highlighting the need for further studies to clarify prevalence, impact, and the potential value of systematic screening.

### 4.4 LVM Regression and LV Reverse Remodeling

Interestingly, in our study, BAV patients did not achieve a high degree of reverse left ventricle remodeling, as did TAV patients, during the one-month follow-up post-TAVR. A significantly smaller LVMi in the TAV than in the BAV was found at the first month after TAVR. Moreover, there were no significant differences in the LVMi between the TAV and BAV throughout the 24-hour or six-month follow-up. The BAV and TAV patients started with similar LVMi and pro-TAVR values in our study, which were comparable in terms of the proportion of LVMi in the two groups. Patients with BAV and TAV had almost identical TAVR procedures and comparable clinical outcomes. We hypothesized that patients with BAV might undergo a different process of left ventricular remodeling after TAVR.

Given that TAVR corrects only the valvular abnormality. In patients with BAV, the absence of direct intervention on the aorta and altered blood flow in the ascending aorta may attenuate short-term improvements in LVMi. It is generally assumed that aortopathy will improve after surgery for aortic morphology; however, a recent study revealed that the response of blood flow to aortic cells differs between BAV and TAV patients. These authors proposed that the differences above between BAV and TAV patients are genetic variance [35].

In addition to genetic theories, the progression of myocardial fibrosis has been found to occur in patients with AVS as a common pathological change. This change is presumed to lead to myocyte apoptosis, subsequent replacement fibrosis and an increase in extracellular volume [36]. LVH is associated with pressure overload induced by AVS [37]. LVH is characterized by increased extracellular collagen content and myocyte hyperplasia, which results in diastolic dysfunction of the left ventricle [38]. Moreover, increased left atrial size is the later consequence of ventricular stiffness [39]. Notably, the more advanced the myocardial fibrosis is, the more abnormal the left ventricular structure is, and the less likely short-term complete remodeling is, especially in BAV patients [40]. It takes years for patients to experience remodeling of myocardial interstitial fibrosis in the late phase of LVM regression. However, myocardial edema might resolve on account of the diffusion of left ventricular pressure overload within a short time during the very early phase of LVM regression. A less pronounced improvement in the LVMi in BAV patients within a brief follow-up period may be associated with more hypertrophy and fibrosis along with the long-term onset of pressure overload, leading to less reversibility [41].

### 4.5 Clinical Implications

The proportion of patients with BAVs undergoing TAVR in China is relatively high, ranging from 40% to 50%, significantly exceeding the rates observed in Western countries (1.6% to 9.3%) and even in other regions of Asia. The use of the TAVR procedure in the Chinese population is now shifting to younger and lower risk patients, among whom younger BAV patients will be more prevalent than older TAV patients [42]. Our findings clearly demonstrated differences in LVMi regression and outcomes between BAV and TAV patients during follow-up, which have important clinical implications not only for doctors but also for patients. Our findings suggest that BAV patients might experience less reverse left ventricle remodeling post-TAVR than patients with TAV during one-month follow-up but similar midterm remodeling during six-month follow-up. TAVR of both types appeared to be safe and effective according to our study. Although there are still challenges associated with using the TAVR in BAV, our study showed that the TAVR procedure could be safely and highly recommended even for BAV patients. As a result, TAVR is expected to increasingly address a growing number of patients with bicuspid AVS worldwide in the future.

As experienced clinicians, we could better predict LVMi regression on account of its favorable clinical outcomes after TAVR as well as how to augment it. First, it would be better to define the problems that are expected to be solved in BAV patients, such as AVS and ascending aortic dilation. In addition, multimodal images, such as echocardiography, MDCT assessment and cardiac magnetic resonance, should be used for accurate diagnosis and specific disease typing and anatomical measurements. Finally, the pathological background should be identified from the basic experimental level to intervene in early risk stratification. Thus, in the current context of advocating precision medical treatment, recognizing the differences in BAV is a prerequisite for formulating various countermeasures.

### 4.6 Limitations

There are several limitations to be addressed when interpreting our results. First, our findings represent a single-center retrospective study, which is an intrinsic limitation of an observational study. Because of the small sample size and limited number of patients, these two groups may not have strong statistical power, although the two groups’ baseline characteristics were comparable. However, firth’s correction was used for revision in our study due to the small sample size. All the data were analyzed using SPSS Version 25.0 (IBM Corp., Armonk, NY, USA). The statistical analysis above may remedy such a deficiency to a large extent. We strongly believe the findings of our study are reliable. Second, it is difficult to draw definitive conclusions with respect to the durability of the TAVR considering midterm follow-up outcomes. We would like to enlarge our cohort and prolong the follow-up in future studies. Third, LVMi was measured by echocardiography, which has lower accuracy than magnetic resonance imaging, but the metrical data were obtained with excellent reproducibility in core laboratories. Fourth, this study compared BAV and TAV without further subclassifying BAV. The Sievers classification was not applied because of its limitations, and this represents a constraint of our work. Future studies will adopt updated classification systems to provide more detailed insights into BAV subtypes. Fifth, the wide CIs observed in some analyses, such as for ARD, likely reflect the limited sample size and variability within the study cohort rather than calculation errors. These findings should therefore be interpreted with caution, and larger studies are needed to validate our results. In addition, magnetic resonance imaging is much more expensive than echocardiography and was not available in study. Finally, the experience accumulated from surgeons needs to be taken into consideration. The size of the valve should be carefully determined based on comprehensive analysis of the landing zone calcification and annular dimension. Complications after TAVR may be influenced by operator discretion, leading to different clinical outcomes.

## 5. Conclusions

Patients with BAV might experience less reverse left ventricle remodeling post-TAVR than patients with TAV during the one-month follow-up but similar midterm remodeling during the six-month follow-up. However, additional studies need to be performed to evaluate the durability of the TAVR procedure in BAV.

## Availability of Data and Materials

The raw data of this article, which support the conclusions, will be made available by the authors, without undue reservation.
